# COVID-19's intra-urban inequalities and social vulnerability in a medium-sized city

**DOI:** 10.1590/0037-8682-0445-2021

**Published:** 2022-04-08

**Authors:** Mário Círio Nogueira, Isabel Cristina Gonçalves Leite, Maria Teresa Bustamante Teixeira, Marcel de Toledo Vieira, Fernando Antonio Basile Colugnati

**Affiliations:** 1 Universidade Federal de Juiz de Fora, Faculdade de Medicina, Departamento de Saúde Coletiva, Juiz de Fora, MG, Brasil.; 2 Universidade Federal de Juiz de Fora, Instituto de Ciências Exatas, Departamento de Estatística, Juiz de Fora, MG, Brasil.; 3 Universidade Federal de Juiz de Fora, Faculdade de Medicina, Departamento de Internato, Juiz de Fora, MG, Brasil.

**Keywords:** COVID-19, Spatial analysis, Social inequality, Urban health

## Abstract

**Background:**

Social conditions are related to the impact of epidemics on human populations. This study aimed to investigate the spatial distribution of cases, hospitalizations, and deaths from COVID-19 and its association with social vulnerability.

**Methods:**

An ecological study was conducted in 81 urban regions (UR) of Juiz de Fora from March to November 2020. Exposure was measured using the Health Vulnerability Index (HVI), a synthetic indicator that combines socioeconomic and environmental variables from the Demographic Census 2010. Regression models were estimated for counting data with overdispersion (negative binomial generalized linear model) using Bayesian methods, with observed frequencies as the outcome, expected frequencies as the offset variable, and HVI as the explanatory variable. Unstructured random-effects (to capture the effect of unmeasured factors) and spatially structured effects (to capture the spatial correlation between observations) were included in the models. The models were estimated for the entire period and quarter.

**Results:**

There were 30,071 suspected cases, 8,063 confirmed cases, 1,186 hospitalizations, and 376 COVID-19 deaths. In the second quarter of the epidemic, compared to the low vulnerability URs, the high vulnerability URs had a lower risk of confirmed cases (RR=0.61; CI95% 0.49-0.76) and a higher risk of hospitalizations (RR=1.65; CI95% 1.23-2.22) and deaths (RR=1.73; CI95% 1.08-2.75).

**Conclusions:**

The lower risk of confirmed cases in the most vulnerable UR probably reflected lower access to confirmatory tests, while the higher risk of hospitalizations and deaths must have been related to the greater severity of the epidemic in the city’s poorest regions.

## INTRODUCTION

In December 2019, the first case of COVID-19 was reported in Wuhan, China. The disease quickly spread worldwide, prompting the World Health Organization to declare a public health emergency of international concern on January 30, 2020. Brazil declared a national public health emergency on February 3, 2020, and on February 25, 2020, had its first confirmed case in the state of São Paulo. As of March 11, 2021, the country had already registered more than 11 million cases and 270 thousand deaths from the disease^1^ ^,^ ^2^.

Geographical and social conditions impact the vulnerability of populations in their territories. The role of characteristics such as race/color/ethnicity, socioeconomic level, and place of residence in the production of differences in the rates of infection and complications of COVID-19 is significant, pointing to health inequity^3^ ^,^ ^4^. Brazil, a continental country with great social heterogeneity, has characteristics that facilitate the spread of the epidemic throughout its territory, although with areas of greater risk of sustainable transmission and greater social impact of the epidemic on the population^5^.

The state of Minas Gerais (MG) has the second largest population in the country, with over 21 million inhabitants. Geographic proximity to major centers and the intense urban mobility of its population have become elements that facilitate the entry and dissemination of COVID-19. After the first confirmed case on March 9, 2020, the epidemic expanded in the following months to most municipalities in the state^6^. Juiz de Fora is the pricipal city of the southeastern health macro-region of the state and the fourth most populous city in the Zona da Mata region, with almost 600 thousand inhabitants. With an Human Development Index (HDI) of 0.778, it has almost 30% of the population with a nominal monthly income per capita of up to 1/2 minimum wage, according to the last demographic census (https://cidades.ibge.gov.br/brasil/mg/juiz-de-fora/panorama). The city had its first confirmed case on March 10, 2020^7^, and according to the web platform “JF Salvando Todos” (http://jfsalvandotodos.ufjf.br/), on June 6, 2021, it had 34,296 cases and 1,657 confirmed by COVID-19 deaths.

In Brazil, there are few analyses of the impact of social vulnerability in urban regions on the number of cases, hospitalizations, and deaths due to COVID-19, considering the differences in age structure between the regions. The spatial analysis approach aims to understand the dynamics of disease occurrence in a community, which can support the planning of local public policies to minimize the impact of inequities.

This study aimed to investigate the spatial distribution of suspected and confirmed cases, hospitalizations, and deaths from COVID-19, adjusted for age, and their association with social vulnerability in the urban area of Juiz de Fora.

## METHODS

### Study population, design, and data sources

**FIGURE 1:**
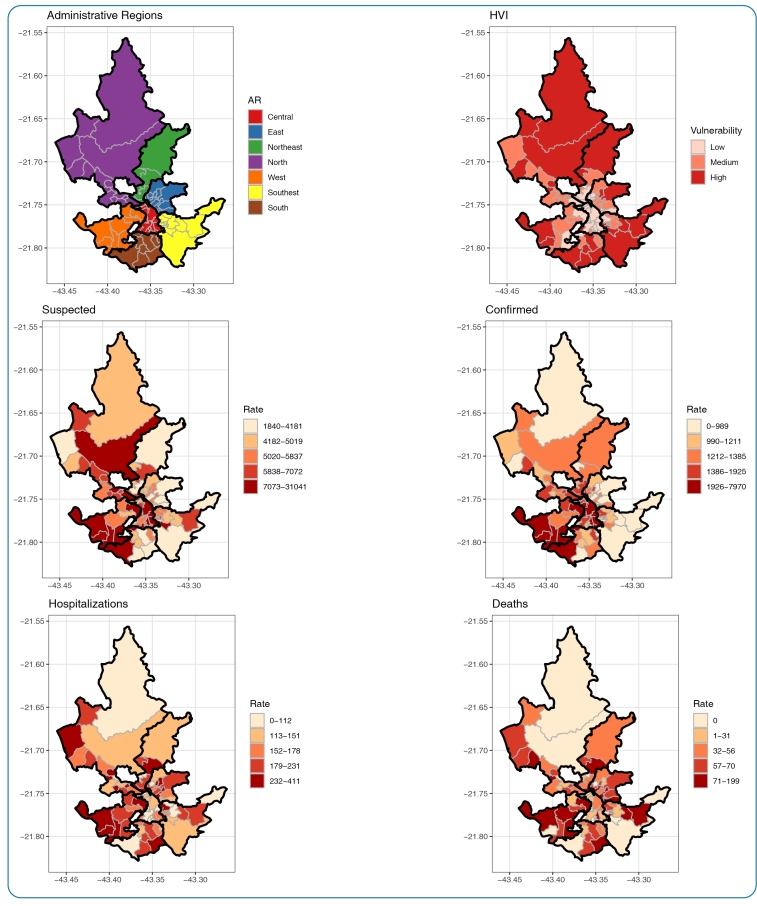
Urban Regions **(UR)** of Juiz de Fora by Administrative Regions **(AR)** and by Health Vulnerability Index **(HVI)** categories, and standardized rates of suspected, confirmed, hospitalizations and deaths by COVID-19 in Juiz de Fora, March to November 2021.

An ecological study was conducted as units of analysis with 81 urban regions (UR) of the city of Juiz de Fora/MG, distributed into seven administrative regions (AR) (Figure 1).

Data on notifications of flu-like syndrome (FS) and hospitalizations for severe acute respiratory syndrome (SARS) in Juiz de Fora were provided by the Municipal Health Department (in Portuguese, SMS/JF) on 12/14/2020. The demographic and socioeconomic data of the URs were obtained from the 2010 Demographic Census. The study period was March to November 2020.

Suspected and confirmed cases, hospitalizations, and deaths due to COVID-19 residing in the urban area of Juiz de Fora, accumulated from March to November 2020, were analyzed. Suspected and confirmed cases were extracted from the FS notification database (selected by notification date between March and November), while admissions and deaths were confirmed by COVID-19 from the SARS notification database (selected by hospital admission date between March and November). For FS notifications, the laboratory test result for COVID-19 was verified until December 14th, and for SARS notifications, the evolution to death was verified until December 28th.

The georeferencing of the data for the URs was performed using the geocode function from the ggmap library of the R program (v. 3.6.1; https://cran.r-project.org/), which consults the geographic coordinates in the Google Maps API.

For the four types of COVID-19 data (suspected cases, confirmed cases, admissions, and deaths), age-standardized rates (per 100,000 population) were estimated for each UR using the new world standard population^8^. In addition, the relative risks (RR_i_) were also estimated as the ratio between the observed (Y_i_) and expected (E_i_) cases in each UR:

$${RR}_{i}=\frac{Y_{i}}{E_{i}}$$

To consider the differences in age structure between the different URs, the expected cases of each UR (E_i_) were estimated as the sum of the expected values in each age group obtained by multiplying the specific rates per age group of the city (r_j_^s^) by the specific population of the age group in each UR (n_j_):

$$E_{i}=\sum_{j=1}^{m}r_{j}^{\left(s\right)}n_{j}$$

### Exposure variable

The Health Vulnerability Index (HVI) was used as a synthetic indicator based on socioeconomic and environmental variables from the 2010 Demographic Census, which has already been used in other studies conducted in Juiz de Fora with health outcomes^9^ ^,^ ^10^. The HVI was calculated as a weighted average of the following variables: percentage of households with inadequate or absent water supply, percentage of households with inadequate or absent sanitary sewage, percentage of households with inadequate or absent garbage disposal, the ratio of residents per household, percentage of illiterate people, percentage of households with per capita income up to ½ minimum wage, the average monthly nominal income of people in charge, and percentage of black, brown, and indigenous people. Based on the HVI score, URs were categorized into three degrees of health vulnerability: low, medium, and high. Medium vulnerability corresponds to HVI values between half a standard deviation below and above the average; low vulnerability corresponds to values below average and high vulnerability above average.

### Descriptive and exploratory analysis

For all exposure and outcome variables, tables were drawn based on their statistical distribution and thematic maps with spatial distributions in the URs.

COVID-19 data were stratified into three periods, each with three months, in order to portray different moments of the epidemic in the city in 2020: initial period of exponential growth, between March and May; intermediate period with fluctuating indicators at high levels, between June and August; and final period that started with a slight reduction but ended with new exponential growth, from September to November.

In the exploratory spatial analysis, global (Moran's I coefficient) and local (LISA - Local Indicator of Spatial Association) spatial autocorrelation measures of all variables were estimated to identify patterns of spatial dependence. LISA, which allows for the decomposition of global indicators into the contribution of each observation, makes it possible to identify areas with their spatial dynamics (clusters), where spatial dependence is even more pronounced, whether in high-risk (high-high) or low-risk (low-low) areas^11^.

### Inferential analysis

Regression models were adjusted for counting data with overdispersion, with estimation using Bayesian methods, with the observed frequency as the outcome, the expected frequency as the offset variable, and the categorized HVI as an explanatory variable. Three mixed generalized linear models were fitted, with a log link function and negative binomial family: a) model only considering all factors as fixed effects; b) the previous model with the inclusion of an unstructured random effect, which can capture the effect of unmeasured factors; and c) model with the inclusion of both the unstructured random effect and a structured spatial effect, which can capture the spatial correlation between observations. The criterion for choosing the best model was the lowest value of DIC (deviance information criterion). The Bayesian estimation method used was the integrated nested Laplace approximation (INLA), using the INLA library (http://www.r-inla.org/) of the R program (https://cran.r-project.org /). Relative risk posteriors are summarized as the mean for point estimates and their respective 95% credibility intervals (CI95%).

The three models are described below.

**Model A:**

$$y_{i}\;\sim \;NB(\mu_{i},\kappa_{i}\left(\theta \right))$$

$$log\;(\mu_{i})=log\;\left(e_{i}\right)\;+log\;\left(\rho_{i}\right)\;=log\left(e_{i}\right)+\beta_{0}+\;\beta_{1}x_{i1}$$

$$\theta =\;\mu +\;{\frac{\mu }{\kappa }}^{2}$$

$$RR=\rho_{i}=e^{\beta_{0}+\beta_{1}x_{i1}}$$

**Model B:**

$$y_{i}|\psi_{i}\;\sim \;NB(\mu_{i},\theta_{i})$$

$$log\;(\mu_{i})=log\;\left(e_{i}\right)\;+log\;\left(\rho_{i}\right)\;=log\left(e_{i}\right)+\beta_{0}+\;\beta_{1}x_{i1}+\;\psi_{i}$$

$$\psi_{i}\;\sim \;N(0,\sigma_{\psi }^{2})$$

$$RR=\rho_{i}=e^{\beta_{0}+\beta_{1}x_{i1}+\psi_{i}}$$

**Model C:**

$$y_{i}|\phi_{i},\upsilon_{i}\;\sim \;NB(\mu_{i},\theta_{i})$$

$$log\;\left(\mu_{i}\right)=log\;\left(e_{i}\right)\;+log\;\left(\rho_{i}\right)\;=log\left(e_{i}\right)+\beta_{0}+\;\beta_{1}x_{i1}+\;\phi_{i}+\upsilon_{i}$$

$$\phi_{i}\;\sim \;N(0,\sigma_{\phi }^{2})$$

$$\upsilon_{i}\;\sim \;CAR(\sigma_{\upsilon }^{2})$$

$$RR=\rho_{i}=e^{\beta_{0}+\beta_{1}x_{i1}+\phi_{i}+\upsilon_{i}}$$

**In which:**

y = observed response variable (cases)

μ = Estimated mean of the response variable

θ = estimated model parameters

e = expected value for the response variable

RR = ρ = estimated relative risk

x = explanatory variable (categorized HVI)

β = coefficients estimated by the model.

σ = variance

ψ = unstructured random effect in Model B

φ = unstructured random effect in Model C

υ = spatial-structured effect

### Ethical considerations

This project was approved by the Research Ethics Committee of the Federal University of Juiz de Fora on 09/02/2020 (CAAE No. 36855920.0.0000.5133).

## RESULTS

There were 30,071 notifications of FS from March to November 2020, of which 8,063 had positive laboratory test results for COVID-19. In the same period, 1,186 hospital admissions were confirmed for COVID-19, of which 376 progressed to death. There was a higher proportion of women for suspected and confirmed cases and a higher proportion of men for admissions and deaths. Cases predominated in the age groups of 20 to 39 years and 40 to 59 years, while hospitalizations and deaths predominated in those over 60 years. Owing to the large proportion of missing data regarding race/color, it is not possible to determine its actual distribution. There were 283 notifications of FS in pregnant women, of which 41 had a laboratory confirmation, but only three hospitalizations and no deaths. The proportion of people at high risk of unfavorable prognosis due comorbidities increased from 22.71% of FS notifications to 90.69% of deaths. Regarding the categories of social vulnerability of the place of residence, a greater proportion of patients in the UR had medium vulnerability. In relation to AR, although the central region had the highest proportion of cases, the east region had the highest proportion of deaths. Most notifications of suspected cases were made in hospital shifts (45.90%) or basic health units (20.85%), whereas confirmed cases were notified in other places, such as clinical analysis laboratories (Table 1).

**TABLE 1:** Characteristics of suspected and confirmed cases, hospitalizations, and deaths due to COVID-19 of residents in the urban area of Juiz de Fora from March to November 2020.

	Suspected		Confirmed		Admissions		Deaths	
Variables	N	%	N	%	N	%	N	%
TOTAL	30071	100.00	8063	100.00	1186	100.00	376	100.00
**Sex**								
Female	17181	57.13	4213	52.25	539	45.45	174	46.28
Male	12881	42.84	3845	47.69	647	54.55	202	53.72
Ignored	9	0.03	5	0.06	0	0.00	0	0.00
**Age group**								
0 to 9 years	1539	5.12	188	2.33	17	1.43	1	0.27
10 to 19 years	1371	4.56	250	3.10	8	0.67	0	0.00
20 to 39 years old	14215	47.27	3694	45.81	114	9.61	10	2.66
40 to 59 years old	9391	31.23	2824	35.02	355	29.93	65	17.29
60 years and older	3555	11.82	1107	13.73	692	58.35	300	79.79
**Race/color**								
White	6927	23.04	1091	13.53	563	47.47	180	47.87
Brown	2542	8.45	250	3.10	186	15.68	64	17.02
Black	2253	7.49	211	2.62	155	13.07	56	14.89
Yellow	170	0.57	32	0.40	15	1.26	2	0.53
Indigenous	11	0.04	2	0.02	0	0.00	0	0.00
Ignored	18168	60.42	6477	80.33	267	22.51	74	19.68
**Pregnant**								
No	29788	99.06	8022	99.49	1183	99.75	376	100.00
yes	283	0.94	41	0.51	3	0.25	0	0.00
**Risk**								
Usual	16205	53.89	4005	49.67	247	20.83	35	9.31
High	6829	22.71	1577	19.56	939	79.17	341	90.69
Ignored	7037	23.40	2481	30.77	0	0.00	0	0.00
**Vulnerability of the Urban Region of residence**								
Low	9777	32.51	3178	39.41	354	29.85	121	32.18
Medium	12832	42.67	3256	40.38	519	43.76	156	41.49
High	7462	24.81	1629	20.20	313	26.39	99	26.33
**Administrative Region of residence**								
Central	7352	24.45	2204	27.33	214	18.04	67	17.82
East	4435	14.75	1146	14.21	238	20.07	79	21.01
Northeast	2658	8.84	770	9.55	125	10.54	45	11.97
North	6945	23.10	1631	20.23	258	21.75	74	19.68
West	2530	8.41	738	9.15	89	7.50	28	7.45
Southeast	3065	10.19	632	7.84	126	10.62	40	10.64
South	3086	10.26	942	11.68	136	11.47	43	11.44
**Location of notification**								
Hospital	13803	45.90	2467	30.60	-	-	-	-
Basic Health Units	6270	20.85	376	4.66	-	-	-	-
Emergency Service	2405	8.00	277	3.44	-	-	-	-
Other	7593	25.25	4943	61.30	-	-	-	-

Of the 81 URs, 27 showed low, 29 medium, and 25 high socio-environmental vulnerability. There was a predominance of low vulnerability in most central URs in the city (Figure 1).

The estimated population in 2020 ranged from 180 to 24,516 in the URs, with an average of 6,580 inhabitants. The HVI also had a wide range, from 0.14 (least vulnerability) to 0.79 (greater vulnerability). Notifications, confirmations, hospitalizations, and deaths showed variation similar to that of the population, with higher values ​​in the most populous URs. From March to November 2020, the average standardized rate was 6,308.02 for suspects, 1,620.46 for confirmed, 168.14 for admissions, and 45.01 for deaths, all per 100,000 population. Apart from hospitalization, the other rates had a significant spatial correlation, although of low magnitude, with a positive correlation of suspected and confirmed cases and a negative correlation of mortality, whereas the HVI had a slightly greater positive spatial correlation. The number and standardized rates of suspected, confirmed, hospitalizations, and deaths from COVID-19 increased every quarter (Table 2).

**TABLE 2:** Distribution of indicators in urban regions (UR) of the municipality and Moran's coefficient I (spatial autocorrelation measure), by period, March to November 2020, Juiz de Fora/MG.

Variables	Average	DP	Min	Q1	Q2	Q3	Max	I*
Population	6579.62	5621.57	180	2833	5074	8118	24516	0.07
HVI	0.32	0.14	0.08	0.20	0.32	0.42	0.79	0.47*
**Suspected**								
Total	371.25	312.40	4	173	276	471	1788	0.06
Period 1	50.27	41.52	1	22	42	67	210	0.04
Period 2	141.78	124.52	2	63	105	182	708	0.07
Period 3	179.20	151.18	1	81	137	243	880	0.07
**Confirmed**								
Total	99.54	89.82	0	40	72	129	494	0.08
Period 1	6.35	6.89	0	2	4	8	37	0.14*
Period 2	42.35	38.53	0	17	30	53	215	0.06
Period 3	50.85	46.85	0	21	39	60	253	0.10
**Hospitalizations**								
Total	14.64	13.05	0	6	11	21	66	0.03
Period 1	1.79	2.43	0	0	1	2	13	-0.07
Period 2	5.65	5.46	0	2	4	8	30	0.07
Period 3	7.20	6.66	0	2	5	10	32	0.05
**Deaths**								
Total	4.64	5.11	0	1	3	7	27	0.02
Period 1	0.56	0.97	0	0	0	1	5	-0.10
Period 2	1.80	2.12	0	0	1	3	9	0.02
Period 3	2.28	2.71	0	0	1	3	13	0.04
**Suspected rate**								
Total	6308.02	4092.20	1840.18	4222.65	5399.49	6796.95	31040.74	0.22*
Period 1	876.12	588.51	83.11	575.06	733.85	936.21	4418.58	0.31*
Period 2	2340.85	1508.19	629.34	1634.97	2035.22	2506.24	12023.41	0.22*
Period 3	3091.05	2160.89	792.72	2085.09	2564.02	3383.26	14598.75	0.18*
**Confirmed rate**								
Total	1620.46	1188.01	0	1090.01	1288.09	1664.96	7970.24	0.27*
Period 1	113.93	154.27	0	46.26	72.99	122.01	961.73	0.26*
Period 2	671.63	536.83	0	439.86	549.07	724.62	4207.92	0.19*
Period 3	834.89	667.78	0	517.44	628.7	947.23	5210.34	0.19*
**Hospitalization rate**								
Total	168.14	86.25	0	124.59	163.74	199.82	410.77	0.05
Period 1	25.67	49.08	0	0	16.57	27.73	396.36	0.04
Period 2	64.53	44.07	0	35.46	64.24	82.03	208.62	-0.04
Period 3	77.94	53.32	0	49.05	68.33	104.43	350.13	-0.10
**Mortality rate**								
Total	45.01	38.91	0	10.88	40.37	68.99	198.62	-0.14*
Period 1	5.63	11.55	0	0	0	7.22	63.19	-0.11
Period 2	16.76	17.58	0	0	14.91	27.27	90.85	0.02
Period 3	22.62	27.67	0	0	15.52	32.91	156.95	-0.15*

*I: Moran's coefficient, significant at the 5% level; **HVI:** Health vulnerability index; **Rate:** Standardized rates per 100,000 people; **SD:** standard deviation; **Min:** minimum; **Q1:** first quartile; **Q2:** median; **Q3:** third quartile; **Max:** maximum; **Period 1:** March to May; **Period 2:** June to August; **Period 3:** September-November.

**FIGURE 2:**
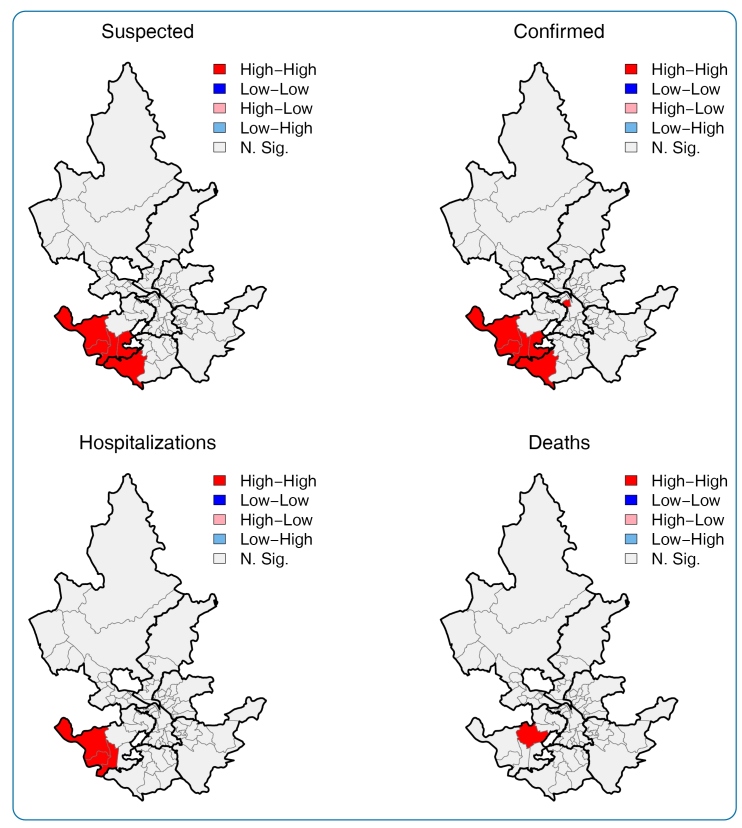
Local spatial autocorrelation **(LISA)** maps for standardized rates of suspected, confirmed, hospitalizations and deaths by COVID-19 from March to November 2020 in Juiz de Fora / MG. **N. Sig.:** not significant.

Regarding the spatial distribution of the indicators in the URs of the city, the standardized rates were higher in the South and West ARs, where UR with high vulnerability predominated. On the other hand, URs located in the city’s central area, with less social vulnerability, had high rates of confirmed cases but low rates of hospitalization and mortality. (Figure 1). The local spatial autocorrelation map (*LISA*) confirmed areas of high rates and neighborhoods with high values in the same AR. Another high-risk cluster of confirmed cases was located in the central region (Figure 2).

**FIGURE 3:**
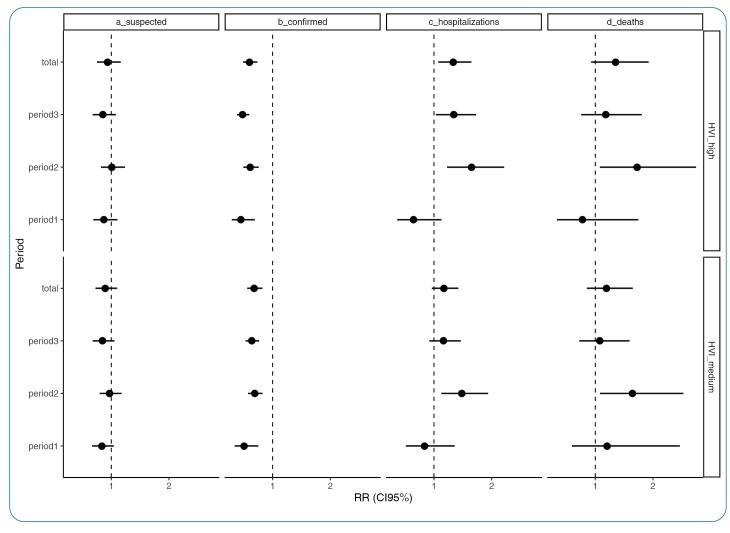
Results of the regression models (relative risk - RR and 95% credible interval - CI95%) between the Health Vulnerability Index **(HVI)** and the risk of suspected cases, confirmed cases, hospitalizations, and deaths from COVID- 19 in residents of the urban area of Juiz de Fora from March to November 2020.

A low vulnerability was used as the reference category for the regression models. Notification of suspected cases was not associated with HVI during any period. The medium-and high-vulnerability URs had a lower risk of confirmed cases in all the periods. The risk of hospitalization had the opposite behavior, with a greater risk in the UR of medium vulnerability in the second period (June to August) and in the UR of high vulnerability in the second and third period (September to November), while hospital deaths also presented an increased risk in the medium and high vulnerability URs, but only in the second period (Figure 3).

## DISCUSSION

This study showed spatial heterogeneity in the risk of notifications, confirmations, hospitalizations, and deaths due to COVID-19 in the urban area of Juiz de Fora, a medium-sized city in Minas Gerais. It also highlighted that this heterogeneity was associated with socio-environmental vulnerability in the city districts, with the lowest vulnerability presenting a greater risk of notifying confirmed cases, while the most vulnerable districts presented a greater risk of hospitalizations and deaths.

During the study period, access to services, reflected in notifications of suspects, did not differ between areas with different levels of vulnerability, while the risk of confirmed cases was lower for the most vulnerable regions, suggesting difficulty in accessing the tests generating underreporting of confirmed cases, especially on the outskirts of the city. The risk of hospitalizations and deaths due to COVID-19, on the other hand, was higher in the most vulnerable regions, which does not seem to be related to underreporting since these cases had priority for testing. The epidemic began in the wealthiest regions of the city, before spreading to the periphery. Thus, in the initial period (March to May 2020), there was no difference in the risk of hospitalization or death according to the vulnerability of the urban regions. In period 2 (June to August 2020), there was an increased risk of hospitalizations and deaths in the most vulnerable regions. In the final period (September to November 2020), the increase in hospitalizations and deaths was more widespread, especially in November, when the second wave started in the city.

In a study that analyzed data from Brazil by states and municipalities between February and October 2020, inequalities during the epidemic associated with social vulnerability were also identified. The epidemic initially had a greater burden in more vulnerable states and municipalities; however, due to more intense social distancing measures in these states, there was better evolution, with lower mortality rates in the final period of the study^12^.

Some studies in Brazilian municipalities have found results similar to those presented here. A study carried out in the city of Santa Maria/RS found that at the beginning of the epidemic, there was a greater concentration of confirmed cases of COVID-19 in more central neighborhoods, and during the epidemic, there was a peripheralization of the epidemic to neighborhoods with greater social deprivation^13^. A study conducted in the districts of São Paulo municipality found that the incidence of COVID-19 was concentrated in clusters of districts with a higher proportion of slums and lower salaries^14^. Rio de Janeiro, the second-most populous city in Brazil with great social inequality, also showed spatial heterogeneity in vulnerability to severe cases of COVID-19, identifying the most vulnerable areas as those with the highest density of residents per household, the highest density of older people, and a higher incidence of tuberculosis^15^.

Less access to diagnostic tests in socially more vulnerable areas was also verified in a study carried out in the metropolitan region of São Paulo, in which confirmation of the disease was positively associated with higher per capita income in the census sector of residence^1^. Vulnerable populations in Brazil have poor access to essential health services in the case of COVID-19, especially low-income communities with a predominance of people of black race/color in the urban peripheries of large cities^16^. In a study carried out with data on hospitalizations for COVID-19 in Brazil between the beginning of the epidemic and mid-May, regional and ethnic-racial inequalities in mortality were evidenced. It was hypothesized that regional inequalities, with higher mortality in the North and Northeast of the country, would be related to a greater burden of comorbidities in regions with lower socioeconomic development, while ethnic-racial inequalities, with higher mortality of black and brown people , would be related to less access to health services^17^. In another study carried out with adults admitted for COVID-19 until the beginning of October 2020, a higher risk of death was also identified in black and brown people, in addition to indigenous people^18^.

Spatial inequalities in health are present in territories marked by vulnerabilities with historical origins updated by new economic, social, and political dynamics. They are manifested by inequalities in the risks of exposure, illness, and aggravation as well as by differentiated access to health actions and services. The current economic moment of globalization accentuates social and health inequalities for several reasons, including the generation of more frequent and intense economic crises and increased structural poverty, resulting from greater competition and selectivity of economic processes. In Brazil, these inequalities are especially important, as Brazil is one of the most unequal countries globally, which has been increasing. Inequality is also manifested in the indicators of the COVID-19 pandemic, whether in incidence, mortality, lethality rates, or access to health services^19^.

Studies carried out in Juiz de Fora on other health outcomes, such as mortality from acute myocardial infarction^20^ and incidence of tuberculosis^9^, also showed a similar pattern of lower rates in the city’s central area and higher rates in more peripheral regions. This is due not only to a compositional effect, as in the central area, there is a greater proportion of people with higher income and education, but also to a contextual effect, as the more peripheral regions are marked by more precarious infrastructure with less access to services in general.

In the US, there are regional inequalities in access to health services, specifically to COVID-19 confirmation tests, which can compromise the control of the epidemic by being related to the undetected spread of the disease^21^. In New York City, a study identified an association between areas with a higher proportion of blacks, Hispanics, and poor people with a higher proportion of positive tests for COVID-19^22^. In the state of Georgia, a set of socioeconomic indicators, which included the percentage of children in poverty and the percentage of adults without health insurance, was associated with the incidence rate of COVID-19^23^. In the first 200 days of the epidemic in US counties, the incidence and mortality rates of COVID-19 were associated with income inequality and ethnic-racial composition^24^. In New York and other urban centers, some vulnerable groups were most affected by the COVID-19 epidemic, such as African Americans, Latinos, immigrants, and native peoples. Some of the structural causes of these inequalities create barriers for these social groups to practice social distancing, such as residential segregation and structural racism^25^. In another study conducted in Chicago, spatial clusters of social vulnerability and risk factors for death from COVID-19^26^ were found. An ecological study showed an association between areas with greater income inequality, as measured by the Gini index, and higher incidence and mortality rates of COVID-19 in the USA^27^.

In European countries, social inequalities related to the COVID-19 pandemic have been identified. A study conducted in Sweden showed that the infection rate was 3-4 times higher in socioeconomically vulnerable areas^28^. In a survey representative of the UK population, social groups with households of more than five people had the greatest impact on their well-being during the epidemic and lockdown measures^29^.

There was an expectation that Brazil could do well in controlling the epidemic, as it has a universal health system centered on a primary care strategy with community agents in the most socially vulnerable areas; in the recent past, it has shown effectiveness in controlling various diseases and in reducing health inequalities^2^. At the beginning of the pandemic, several measures were implemented, including the declaration of a national public health emergency in January 2020, activation of an emergency health operation center, and recommendation of health surveillance measures and social distancing^30^. However, this expectation did not materialize, since Brazil has become one of the countries with the highest incidence and mortality from COVID-19. A characteristic of the Brazilian response to the pandemic was the lack of coordination by the federal government during its course, which still promoted ineffective interventions. The epidemic rapidly spread to the country’s interior, with some differences between states. Some factors that could be related would be large disparities in economic and health resources; the great communication between municipalities and regions in terms of transport, services, and trade; the alignment of some state and municipal government officials with the conduct of the federal government in denying the importance of social distancing measures; the circulation of the virus was not detected early due to the difficulties of well-equipped health surveillance and the lack of coordination, coherence, and criteria in state and municipal decisions about the closing and opening moments of non-essential activities^2^.

This study showed that, in medium-sized cities, various aspects of the behavior of the COVID-19 epidemic are closely related to the life context of population groups. Although the public health services network has guaranteed equity in access to first care, reflected in the notification of suspected cases, it has not brought the same guarantee for access to examinations needed to confirm the disease. More importantly, social vulnerability was associated with a higher risk of severe disease progression, thus unveiling the social inequality of the impact of this pandemic on the population. The planning of disease prevention and control actions, not only COVID-19 but also other communicable or noncommunicable diseases, should consider territorial-based epidemiological surveillance so that public policy interventions can reduce social inequalities in health.
